# Improving Older Adult Psychiatry Clerking Process in Aneurin Bevan University Health Board

**DOI:** 10.1192/bjo.2026.11439

**Published:** 2026-06-30

**Authors:** Gianluca Mosaico, Mohamed Elsout, Nabeela Rafiq, Andreas Lappas

**Affiliations:** 1 Aneurin Bevan University Health Board, Newport, United Kingdom; 2 Cwm Taf Morgannwg University Health Board, Bridgend, United Kingdom; 3 Aneurin Bevan University Health Board, Caerphilly, United Kingdom

## Abstract

**Aims:**

Psychiatry resident doctors in Aneurin Bevan University Health Board (ABUHB) reported difficulty locating and completing the paper clerking proforma in use in Older Adult Psychiatry wards, alongside inconsistent electronic availability of clerking information due to delays in scanning and uploading. In this project, we aimed to improve the completeness and accessibility of admission clerking for inpatients in ABUHB Older Adult Psychiatry services, while reducing printed paperwork. In order to achieve this, we introduced a new digital clerking proforma aligned with relevant Royal College of Psychiatrists recommendations and NICE guidance. We hypothesised that introducing a digital proforma would increase overall item completion from 50% to ≥80%.

**Methods:**

This quality improvement project used three Plan–Do–Study–Act (PDSA) cycles. Baseline retrospective data covered 14 consecutive admissions (n=14). The primary outcome was the percentage completion per patient on a 16-item proforma with equal weighting. Process measures captured completion within the History (7 items), Assessment (5 items), and Plan (4 items) domains. Post-baseline data were collected prospectively for consecutiveadmissions during each cycle (n=10 in PDSA-1, n=12 in PDSA-2, n=34 in PDSA-3). In PDSA-1, a new Word proforma was introduced; in PDSA-2, accessibility was improved via shared-drive access; and PDSA-3 involved embedding the proforma to the electronic system, and also aimed at comparing clerkings completed using the electronic proforma with those completed using the previous method.

**Results:**

Median overall completion improved from 55.4% at baseline to 83.9% after PDSA-1, and 87.5% after PDSA-2, remaining stable at 85.7% in PDSA-3. In PDSA-3, completion reached a median of 100% when the electronic proforma was used, versus 53.6% with paper-based clerking.

**Conclusion:**

The introduction of a practical and accessible digital proforma improved clerking completeness, with cost savings and sustainability benefits. Next steps include targeted teaching for resident doctors and senior clinicians, improving proforma usability, ongoing monitoring, and potential spread to other directorates.

